# A serological framework to investigate acute primary and post-primary dengue cases reporting across the Philippines

**DOI:** 10.1186/s12916-020-01833-1

**Published:** 2020-11-27

**Authors:** Joseph R. Biggs, Ava Kristy Sy, Oliver J. Brady, Adam J. Kucharski, Sebastian Funk, Mary Anne Joy Reyes, Mary Ann Quinones, William Jones-Warner, Yun-Hung Tu, Ferchito L. Avelino, Nemia L. Sucaldito, Huynh Kim Mai, Le Thuy Lien, Hung Do Thai, Hien Anh Thi Nguyen, Dang Duc Anh, Chihiro Iwasaki, Noriko Kitamura, Lay-Myint Yoshida, Amado O. Tandoc, Eva Cutiongco-de la Paz, Maria Rosario Z. Capeding, Carmencita D. Padilla, Julius Clemence R. Hafalla, Martin L. Hibberd

**Affiliations:** 1 Department of Infection Biology, Faculty of Infectious and Tropical Diseases, London School of Hygiene and Tropical Medicine, London, UK; 2 Department of Virology, Research Institute for Tropical Medicine, Manila, Philippines; 3 Dengue Study Group, Research Institute for Tropical Medicine, Manila, Philippines; 4 Department of Infectious Disease Epidemiology, Faculty of Epidemiology and Population Health, London School of Hygiene and Tropical Medicine, London, UK; 5 Centre for the Mathematical Modelling of Infectious Diseases, London School of Hygiene and Tropical Medicine, London, UK; 6 Philippine Epidemiology Bureau, Department of Health, Manila, Philippines; 7 Pasteur Institute of Nha Trang, Nha Trang, Vietnam; 8 National Institute of Hygiene and Epidemiology, Hanoi, Vietnam; 9 Paediatric Infectious Diseases Department, Institute of Tropical Medicine, Nagasaki University, Nagasaki, Japan; 10 Institute of Human Genetics, National Institute of Health, University of the Philippines, Manila, Philippines; 11 Philippine Genome Centre, University of the Philippines, Manila, Philippines

**Keywords:** Dengue, Flavivirus, Primary, Post-primary, Immuno-epidemiology, Surveillance, Serology, Philippines

## Abstract

**Background:**

In dengue-endemic countries, targeting limited control interventions to populations at risk of severe disease could enable increased efficiency. Individuals who have had their first (primary) dengue infection are at risk of developing more severe secondary disease, thus could be targeted for disease prevention. Currently, there is no reliable algorithm for determining primary and post-primary (infection with more than one flavivirus) status from a single serum sample. In this study, we developed and validated an immune status algorithm using single acute serum samples from reporting patients and investigated dengue immuno-epidemiological patterns across the Philippines.

**Methods:**

During 2015/2016, a cross-sectional sample of 10,137 dengue case reports provided serum for molecular (anti-DENV PCR) and serological (anti-DENV IgM/G capture ELISA) assay. Using mixture modelling, we re-assessed IgM/G seroprevalence and estimated functional, disease day-specific, IgG:IgM ratios that categorised the reporting population as negative, historical, primary and post-primary for dengue. We validated our algorithm against WHO gold standard criteria and investigated cross-reactivity with Zika by assaying a random subset for anti-ZIKV IgM and IgG. Lastly, using our algorithm, we explored immuno-epidemiological patterns of dengue across the Philippines.

**Results:**

Our modelled IgM and IgG seroprevalence thresholds were lower than kit-provided thresholds. Individuals anti-DENV PCR+ or IgM+ were classified as active dengue infections (83.1%, 6998/8425). IgG− and IgG+ active dengue infections on disease days 1 and 2 were categorised as primary and post-primary, respectively, while those on disease days 3 to 5 with IgG:IgM ratios below and above 0.45 were classified as primary and post-primary, respectively. A significant proportion of post-primary dengue infections had elevated anti-ZIKV IgG inferring previous Zika exposure. Our algorithm achieved 90.5% serological agreement with WHO standard practice. Post-primary dengue infections were more likely to be older and present with severe symptoms. Finally, we identified a spatio-temporal cluster of primary dengue case reporting in northern Luzon during 2016.

**Conclusions:**

Our dengue immune status algorithm can equip surveillance operations with the means to target dengue control efforts. The algorithm accurately identified primary dengue infections who are at risk of future severe disease.

**Supplementary information:**

**Supplementary information** accompanies this paper at 10.1186/s12916-020-01833-1.

## Background

Dengue has become the most significant disease-causing arbovirus in the tropical and subtropical world. According to World Health Organization (WHO) global figures, notified cases of dengue have increased 30-fold in the past 5 decades [1], and a further 247,000 suspected dengue cases in the Western Pacific region were reported in 2014 compared to 2008 [2]. This reporting likely grossly underestimated true numbers given the range of dengue clinical manifestations and variable healthcare infrastructures in endemic countries. Instead, modelled estimates approximate 390 million annual dengue cases occur globally, of which 75% are asymptomatic [3]. Dengue emergence is believed to be attributed to rapid population growth, urbanisation, human migration, climate change, and is unhampered by costly control interventions [1].

Infection with one of the four known immunologically distinct dengue virus serotypes (DENV1–4) causes a delayed increase in viremia, combined with potential fever, which decreases within days. This is followed by an increase in immunoglobulin M (IgM) that wanes over months [4]. During a primary infection, immunoglobulin G (IgG) increases during the convalescent stage of disease and persists for life, rendering individuals immune to homologous but not heterologous dengue virus serotypes. Upon a post-primary (secondary, tertiary, quaternary) infection with a contrasting serotype, IgM resurgence is subdued while pre-circulating, non-neutralising IgG increases rapidly with viremia [5]. This enhanced level of non-protective IgG is believed to facilitate rapid viral replication in hosts through an antibody-mediated enhancement (ADE) process [6, 7]. Dengue symptoms range from asymptomatic to severe [3, 8]. According to WHO guidelines, severe symptoms include critical plasma leakage, haemorrhage and organ impairment [9]. These symptoms are thought to arise from host-mediated cytokine storms that occur in response to viral replication [10, 11], and as a consequence, post-primary dengue infections are major risk factors for developing severe disease [12–14].

The gold standard serological method for determining a dengue infection, including whether it is primary or post-primary dengue, remains the WHO haemagglutination inhibition assay (HIA) using acute and convalescent paired sera. A fourfold increase in IgG titre that exceeds or falls below 1:2560 during convalescence is indicative of secondary and primary infections, respectively [15]. Despite the high-throughput nature of this technique, the need for paired sera, collected at least 7 days apart, makes it undesirable for large-scale epidemiological surveillance. To overcome this, commercial IgM and IgG capture ELISAs, used concurrently, can distinguish primary and secondary dengue using a single acute-stage serum sample. For instance, Panbio® capture ELISAs (Alere, Brisbane, Australia, Cat. No.: 01PE10/01PE20) state their IgG seroprevalence threshold of 22 panbio units corresponds to a HAI 1:2560 IgG titre. Therefore, individuals assayed using these kits who are IgM+/IgG− and IgM+/IgG+ supposedly represent primary and secondary dengue infections, respectively.

For dengue surveillance purposes, IgM and IgG capture ELISAs are an affordable and logistically simple way to investigate epidemiological patterns in primary and post-primary dengue. However, this technique is not without caveats. First, given the delay in eliciting anti-DENV IgM following infection, it remains unknown whether early stage, non-immunogenic (IgM− and IgG−), primary dengue cases are detectable using this solely serological diagnostic. Second, given a recent study highlighted primary dengue infections can elicit high IgG levels during the febrile period [16], commercially provided IgG thresholds may misclassify acute primary and post-primary dengue infections. The incorporation of commonly used molecular (PCR) tools may improve the diagnostic capability of this algorithm. In addition, IgG:IgM ratios have been proposed as useful metrics for categorising dengue immune status given that major differences between IgG and IgM occur during post-primary, compared to primary, infections [17, 18]. However, the practical application of these thresholds during the febrile stage of infection is limited [16], suggesting that further studies investigating the stage of infection at which ratios become appropriate, if at all, are warranted.

In dengue-endemic countries including the Philippines, optimising the use of passively collected dengue case report data could strengthen disease surveillance and control. In the Philippines, laboratory-based surveillance efforts currently include routine molecular characterisation of dengue, using sera collected from a representative sample of all case reports. This allows surveillance operations to monitor spatio-temporal dengue serotype patterns across the Philippines. However, molecular characterisation alone does not indicate whether case reports experienced primary or post-primary dengue infections, information that may prove useful in identifying populations at risk of severe symptoms. The aim of this study was to develop a novel dengue immune status algorithm using routinely collected serological and molecular metrics and compare its performance with commercial and WHO-approved practice. The co-circulation of other arboviruses across the Philippines however, which present with similar acute clinical manifestations, including Chikungunya [19, 20], Japanese encephalitis [21] and more recently Zika in 2016 [22], poses a challenge to this effort. Numerous studies have demonstrated antibody responses against dengue virus cross-react with Zika virus [23–25], making it difficult to detect the true causative agent of infection. Upon validation of an appropriate immune status algorithm, we investigated immuno-epidemiological patterns of dengue transmission across the Philippines in 2015/2016 to inform surveillance operations and targeted disease control.

## Methods

### Dengue surveillance in the Philippines

The Philippines, consisting of 7641 islands spanning more than 300,000 km^2^, is one of the countries in the Western Pacific region most heavily burdened by dengue [2]. According to the country’s Department of Health (DOH), all four serotypes of dengue (DENV1–4) co-circulate in the country and reported cases increased from 213,930 to 220,518 between 2015 and 2016, respectively [26]. In 2008, the Philippine Integrated Disease Surveillance and Response (PIDSR) system was established to synchronise and strengthen disease surveillance across the country resulting in dengue becoming notifiable across all Filipino disease-reporting units (DRUs), ranging from local barangay health facilities to major regional hospitals [27]. According to the 2009 WHO criteria [9], the PIDSR categorises patients as having no warning signs, warning signs or severe dengue symptoms. Warning signs include a sudden acute illness coupled with either abdominal pain, vomiting, fluid accumulation, mucosal bleeding, lethargy, liver enlargement, increased haematocrit and/or decreased platelet counts. Severe symptoms include a sudden acute illness coupled with either severe plasma leakage, severe bleeding and/or severe organ impairment.

### Data collection, management and laboratory methods

Serum samples were collected from suspected dengue cases that reported to DRUs (health facilities) across the Philippines according to PIDSR criteria: a previously well person with a 2–7-day prolonged febrile illness coupled with two additional non-specific dengue symptoms. Infants under the age of 6 months were excluded from the study due to the potential persistence of maternal anti-DENV antibodies. A total of 20 sentinel and 185 non-sentinel DRUs across the Philippines participated in the study during 2015 and 2016. Sentinel DRUs supplied 5 random samples per week and included major regional hospitals. Non-sentinel DRUs included any health facility that reported a marked increase in dengue cases/deaths according to PIDSR criteria [27] and supplied samples during these outbreak periods. In total, 10,137 individuals supplied serum to the National Reference Laboratory for Dengue and Other Arboviruses at the Research Institute for Tropical Medicine (RITM), the research arm of the DOH, for further study. Coupled with sera were epidemiological data consistent with the PIDSR system including age, sex, date of birth, date of admission, date of illness onset, symptoms (no warning signs, warning signs and severe), outcome (dead and alive) and DRU address and GPS coordinates. Additionally generated variables include disease day (date of admission–date of illness onset), IgG:IgM ratio (IgG panbio units/IgM panbio units), DRU elevation (metres) and DRU population density (km^2^). DRU-level covariates were generated using 100 m resolution Philippine elevation and population density raster data from 2015 (USGS; earth explorer; USA). Raster values were assigned to the midpoint of the DRUs using corresponding GPS coordinates in ArcGIS (v.10.5).

To focus this study on acute (febrile) dengue cases, those who reported more than 5 days post the onset of febrile symptoms (1318/10,137) or had missing onset/reporting date data (154/10,137) were excluded from the study. Subsequently, those with incomplete serological/molecular data (131/8665) or symptom data (176/8665) were excluded from the final dataset (Additional file 1). Those with missing serological/molecular data were excluded as our algorithm utilises both molecular and serological metrics. To assess whether excluding those with missing data among the febrile surveillance dataset introduced selection bias, we compared percentage demographic characteristics of the final febrile dengue surveillance and those with missing serological/molecular and symptom data. To investigate whether anti-DENV responses cross-react with Zika, a random subset of serum samples from the final 2016 febrile dengue surveillance dataset (1000/3921) were selected for anti-ZIKV IgM and IgG assay.

Serum samples were stored at − 80 °C prior to molecular and serological assay. Among all viable collected samples, dengue viremia was determined using a fourplex real-time polymerase chain reaction (PCR) assay as previously described [28]. In short, dengue serotype-specific primers detect then amplify dengue RNA in serum to determine viremia. Samples were considered PCR positive or negative for dengue if they had critical threshold cycle (Ct) values below or above 36, respectively. To detect the presence of anti-dengue IgG and IgM, samples were assayed using Panbio® capture IgM and IgG ELISA kits (Cat. No.: 01PE10/01PE20, Alere, Brisbane, Australia). Briefly, kits encompass antigen capable of capturing host antibody specific to all four dengue serotypes and include plate-specific calibrators that normalise output optical density (OD) readings to generate standardised antibody panbio units. Pre-determined panbio unit serological thresholds categorised individuals as negative (IgM ≤ 9, IgG ≤ 18), equivocal (IgM 9–11, IgG 18–22) and positive (IgM ≥ 11, IgG ≥ 22) for dengue infections. Consistent with kit specifications, algorithm 1 (A1) classified primary and post-primary dengue cases as being IgM+, IgG− and IgM+, IgG+, respectively. Among samples selected for ZIKV antibody testing, samples were assayed using Euroimmune™ (Lübeck, Germany) ZIKV IgM-ELISA (El 2668–9601 M) and IgG-ELISA (El 2668–9601 G) kits according to specification instructions. The semi-quantitative ratio outputs from these tests were used to dichotomise individuals as anti-ZIKV IgM/IgG positive (OD ratio > 1.1) or negative (OD ratio < 1.1).

### Serological modelling and algorithm validation

Mixture models were used to (1) establish true anti-dengue IgM and IgG seroprevalence and (2) determine, disease day-specific, IgG:IgM ratio thresholds that distinguish primary from post-primary dengue infections. All models were fitted by maximum likelihood with lognormal distributions using the command ‘fmm:glm’ in STATA (v.15, Texas, USA). For IgM and among the entire study population, models were fitted with 3 components to represent the seronegative, primary and post-primary populations. For IgG and among non-active DENV cases (PCR− and IgM−), the models were fitted with 2 components to characterise distributions of those with/without prior IgG exposure to DENV. We compared these models based on a single distribution models using Akaike information criterion (AIC). Lower AIC indicates better model fit. IgM and IgG seroprevalence thresholds refer to the lowest antibody titre values with a classification probability of being seropositive>seronegative.

To determine the primary and post-primary dengue immune status of active dengue cases, disease day-stratified IgG:IgM ratio distributions were fitted with 2-component mixture models to classify the distinct primary and post-primary subpopulations. For each disease day, we calculated IgG:IgM ratio thresholds corresponding to the lowest ratio value with a classification probability of being post-primary>primary. Active dengue cases with ratios above and below these disease day-specific thresholds were categorised as post-primary and primary, respectively. To determine whether IgG:IgM ratios were appropriate to distinguish immune status on specified disease days, we justified the existence of two rather than one ratio distribution using Akaike information criterion. Only 2-component models with lower AIC values compared to 1-component models were used to generate ratio thresholds for specific disease days.

To validate the commercial and novel dengue immune status algorithms, we utilised paired sera from community household members of reporting DENV RDT NS1+ patients involved in a study conducted in Nha Thang, Vietnam. Twenty-one household members reported day of fever and supplied acute and convalescent sera. Paired sera were assayed for anti-DENV NS1 (Rapid diagnostic test, Bio-Rad, France), IgM and IgG using Panbio® capture ELISA kits (as described previously). Using single acute serum samples from household members, dengue immune status was determined according to Panbio® specifications (A1) and our novel algorithm (A2). In addition, using paired sera from household members, dengue immune status was also established corresponding to WHO guidelines [22] (as described previously). The serological agreement of both A1 and A2 to the gold standard WHO technique was used to verify algorithm performance for further use characterising immuno-epidemiological trends in dengue transmission across the Philippines.

To investigate dengue transmission intensity across the Philippines, we estimated anti-DENV IgG seroconversion rates (SCRs) among those reporting with non-active dengue infections (PCR− and IgM−). SCRs, which correspond to the average annual rate individuals seroconvert from anti-DENV IgG− to IgG+, were obtained from IgG age-seroprevalence curves fitted using simple and reversible catalytic models. Assuming individuals seroconvert solely from IgG seronegative to seropositive status, Eq. 1 estimates the probability of being IgG seropositive at specified ages (a) by fitting a constant force of infection parameter (**λ**) by least squares according to the function:

1 $$ \mathbf{P}\left(\mathbf{a}\right)=\left[\mathbf{1}-{\mathbf{\exp}}^{-\boldsymbol{\uplambda} \mathbf{a}}\right] $$

Given immunological protection may decay over time resulting in reporting non-active dengue cases reverting to IgG seronegative status according to our mixture model threshold, Eq. 2 fits an additional constant seroreversion parameter (ρ), by least squares, according to the function:

2 $$ \mathbf{P}\left(\mathbf{a}\right)=\frac{\boldsymbol{\uplambda}}{\boldsymbol{\uplambda} +\boldsymbol{\uprho}}\left[\mathbf{1}-{\mathbf{\exp}}^{-\left(\boldsymbol{\uplambda} +\boldsymbol{\uprho} \right)\mathbf{a}}\right] $$

Likelihood ratio tests were used to determine which model, simple or reversible, best characterised age-IgG seroprevalence data (*p* value < 0.05). All models were fitted by maximum likelihood using a constrained/unconstrained ‘revcat’ command in STATA (v.15).

To investigate the risk factors associated with presenting as a post-primary, rather than a primary, dengue case, we calculated unadjusted odds ratios from a univariable logistic regression model using the ‘logit’ command in STATA (v.15). Explanatory variables included age, sex, disease day, clinical manifestation, DRU elevation and DRU population density.

## Results

### Data description

Between 2015 and 2016, 8665 serum samples were collected from consenting febrile, suspected dengue cases among DRUs across the Philippines, in which 131/8665 and 176/8665 had missing molecular/serological and symptom data, respectively (Additional file 1). Similar demographic characteristics were observed between febrile dengue cases with complete data and those with incomplete molecular/serological and symptom data (overlapping 95% CIs) (Additional file 2). In the final complete dengue surveillance dataset used in this study, demographic information reveals that a slightly higher percentage were male (52.5%), whereas most were aged between 6 and 15 years (44.1%), reported with dengue-like symptoms (69.5%) and reported 3–4 days post the onset of fever (60.5%). Mortality was low among the study population with only 0.4% reported as having died from dengue (Additional file 2).

### Determining dengue immune status

**Fig. 1:**
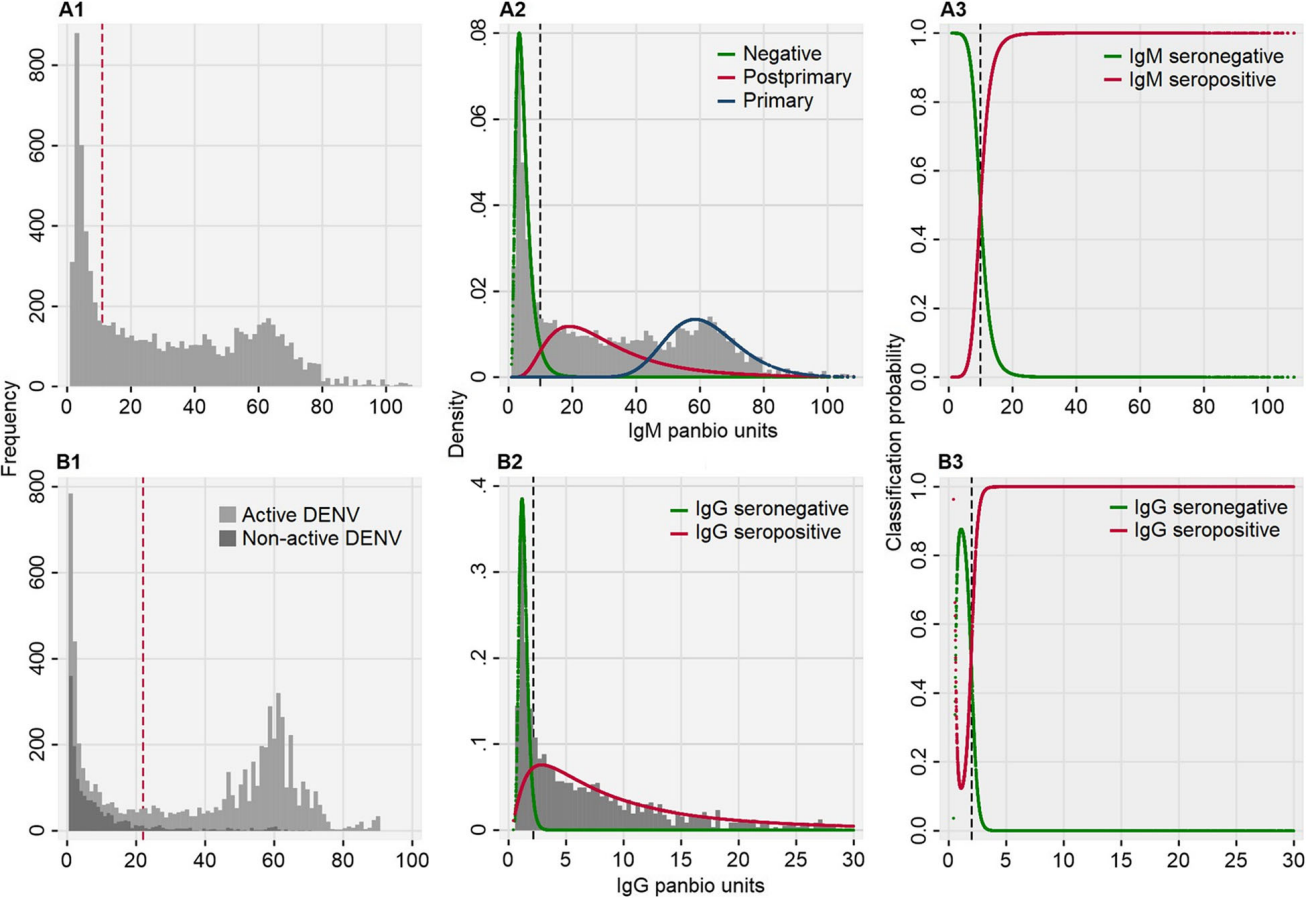
Determining anti-DENV IgM and IgG seroprevalence among the study population. **A1** Histogram of study population anti-DENV IgM panbio units. Red dash: Panbio® seroprevalence threshold (11 panbio units). **A2** Mixture model of anti-DENV IgM panbio units with 3 components representing IgM seronegative, primary and post-primary subpopulations. **A3** Modelled classification probability curves of being anti-DENV IgM seronegative or seropositive. Black dash: lowest IgM panbio unit with a classification probability of being seropositive>seronegative (9.9 panbio units). **B1** Histogram of anti-DENV IgG panbio units stratified by active/non-active DENV status. Red dash: Panbio® seroprevalence threshold (22 panbio units). **B2** Mixture model of anti-DENV IgG titres among non-active DENV cases with 2 components representing IgG seronegative and seropositive subpopulations. **B3** Modelled classification probability curves of being anti-DENV IgG seronegative or seropositive. Black dash: lowest IgG panbio units with a classification probability of being seropositive>seronegative (2.2 panbio units)

Upon re-assessing anti-DENV IgM seroprevalence, we identified a large proportion of the study population had elevated anti-DENV IgM titres resulting in a distribution best characterised by a 3-component, rather than a 1-component, mixture model (AIC difference − 221.2) (Additional file 3). This model provided an anti-DENV IgM seropositivity threshold of 9.9 panbio units, resulting in an IgM seroprevalence of 71.8% (6050/8425) in our population (Fig. 1A). To investigate whether our anti-DENV IgM seroprevalence threshold is representative of all ages, among those aged between 0–5, 6–15, 16–30 and 31+ years, we estimated narrow-ranging anti-DENV IgM seroprevalence thresholds of 9.8, 10.1, 10.3 and 9.7 panbio units, respectively (Additional file 4). Given anti-DENV IgM responses shortly succeed viremia during a dengue infection, we concluded those either PCR+ for DENV RNA or anti-DENV IgM+ represent active dengue cases (83.1%, 6998/8425) while those anti-DENV PCR− and anti-DENV IgM− represent non-active dengue cases (misdiagnoses 16.9%, 1427/8425).

To re-assess anti-DENV IgG seropositivity, among non-active dengue cases, we assumed two subpopulations of those with/without previous IgG exposure to dengue. Rationale supported by the fact a proportion of non-active dengue cases had elevated anti-DENV IgG (Fig. 1B) resulting in a 2-component, rather than a 1-component, mixture model better characterising the IgG panbio unit distribution (AIC difference − 97.7) (Additional file 5). Furthermore, a higher proportion of older non-active dengue cases had elevated IgG compared to younger non-active dengue individuals (Additional file 4). A trend likely attributed to older individuals having a higher probability of being infected with a previous dengue infection prior to reporting than younger individuals. By fitting a 2-component mixture model to the IgG panbio unit distribution of active dengue cases, this yielded a IgG seroprevalence of 2.2 panbio units; non-active dengue cases with IgG panbio units above and below this value were categorised as having historical (69.4%, 991/1427) and negative (30.6%, 436/1427) dengue exposure, respectively. Compared to kit-defined thresholds, modelled anti-DENV IgM and IgG thresholds were 1.1 and 19.8 panbio units lower, respectively.

**Fig. 2:**
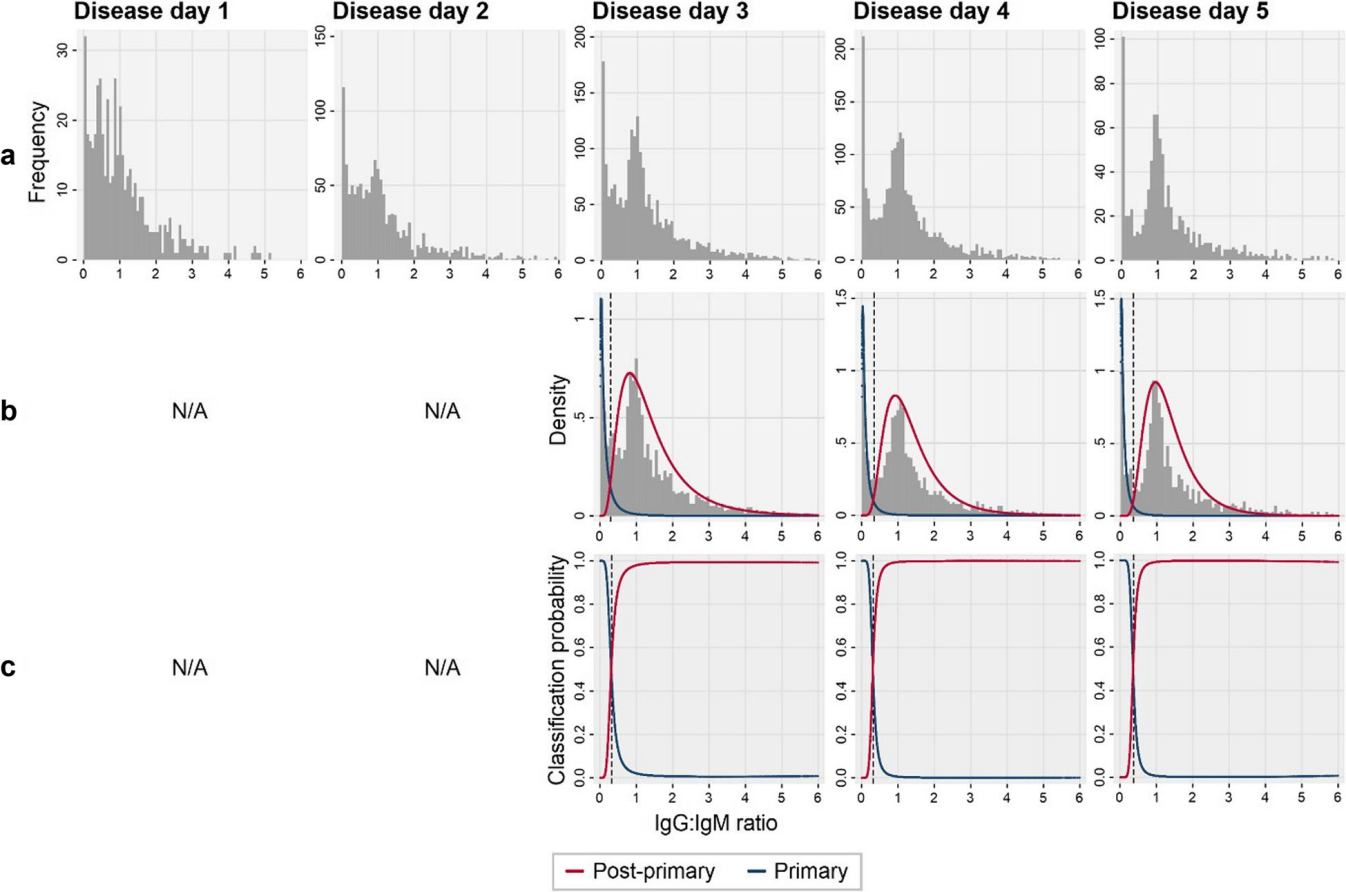
Determining primary and post-primary DENV immune status among active DENV cases. **a** Histogram of individual IgG:IgM ratios among active DENV cases by disease day. **b** Mixture model of IgG:IgM ratio distributions with 2 components representing primary and post-primary subpopulations. **c** Modelled classification probability of being a primary or post-primary DENV case by disease day. Black dash: lowest ratio value with a classification probability of being post-primary>primary (Dd3, 0.44; Dd4, 0.44; Dd5, 0.47). N/A, no statistical justification for two mixture model components (1-component model AIC<2-component model AIC); Dd, disease day

**Fig. 3:**
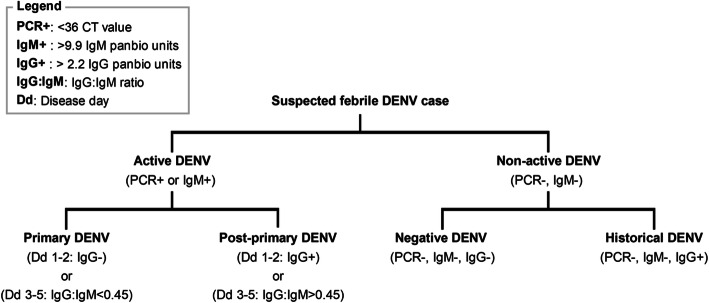
Algorithm 2 (A2): dengue immune status algorithm. Criteria used to determine the dengue immune status of the study population across the Philippines during 2015–2016. Primary DENV, current dengue infection with no previous flavivirus infection; post-primary DENV, current dengue infection with at least one previous flavivirus infection

Among active dengue cases, we determined primary and post-primary dengue immune status by investigating functional, disease day-specific, IgG:IgM ratio distributions (Fig. 2). With increasing disease day (1 to 5), we observed two increasingly distinct lower and higher ratio subpopulations consistent with predicted primary and post-primary dengue infections, respectively (Fig. 2a). These distributions were best fit by a 1-component mixture model on disease days 1 and 2, and a 2-component mixture models on disease days 3–5 (Additional file 6). For disease days 3 to 5, IgG:IgM ratio thresholds, corresponding the lowest ratio with a classification probability of being post-primary>primary, equated to 0.44, 0.44 and 0.47, respectively (Fig. 2b, c). Given the similarity between thresholds, disease day 3–5 ratio thresholds were averaged (0.45) and incorporated into algorithm 2 to distinguish primary and post-primary dengue infections. Active dengue cases on disease days 3–5 with IgG:IgM ratios above and below 0.45 were categorised as post-primary and primary dengue, respectively. For disease days 1–2, with no statistical justification for the existence of two distinct primary and post-primary ratio distributions (1-component AIC<2-component AIC), we opted to determine dengue immune status using the previously calculated IgG seroprevalence threshold. Active dengue cases on disease day 1 or 2 with IgG panbio units above and below 2.2 were categorised as post-primary and primary, respectively. An outline of algorithm 2 (A2) is summarised in Fig. 3 while A1 and A2 study population categorisation is shown in Table 1.

**Table 1:** Dengue immune status categorisation of the study population

DENV immune status	Algorithm			
	1		2	
	*n*	%	*n*	%
Primary	1285	15.3	1576	18.7
Post-primary	4177	49.6	5414	64.3
Historical	–	–	991	11.8
Negative	–	–	436	5.2
Unclassifiable	2963	35.2	8	0.1

Algorithm 1 (A1): Panbio® commercial algorithm. Algorithm 2 (A2): novel algorithm generated in this study

After generating our novel dengue immune algorithm (A2), we compared it to commercial practice (A1). A2 assigned dengue immune status to an additional 35.1% (2955/8425) of the study population who were unclassifiable according to A1 (Table 1). Among the 21 household fever cases, A2 categorised the immune status of all members while A1 only classified 9/21 individuals. Subsequently, we investigated how well each algorithm categorised the immune status of household fever cases with paired sera according to the WHO gold standard method. A2 and A1 achieved 90.5% (19/21) and 71.4% (15/21) serological agreement, respectively (Additional files 7 and 8). These results demonstrate the superiority of A2 compared to A1 and justified its use for investigating immuno-epidemiological patterns of dengue immune status across the Philippines.

Lastly, to assess whether humoral responses against dengue were attributed to other flaviviruses, we investigated anti-ZIKV and anti-DENV cross-reactivity among those categorised as primary and post-primary according to A2 (Additional file 9). Among both primary and post-primary dengue infections, anti-ZIKV IgM responses were low and only 0% (0/154) and 1% (5/508) were IgM seropositive, respectively, according to Euroimmune specifications. This suggests very few of the active DENV infections were recent ZIKV infections. In contrast, among post-primary infections, anti-ZIKV IgG responses were elevated with 23% (118/508) seropositive to anti-ZIKV IgG according to Euroimmune kit instructions. Together, these results suggest post-primary cases include current dengue infections with potential, historical, ZIKV exposure (Fig. 3).

### Dengue transmission dynamics

**Fig. 4:**
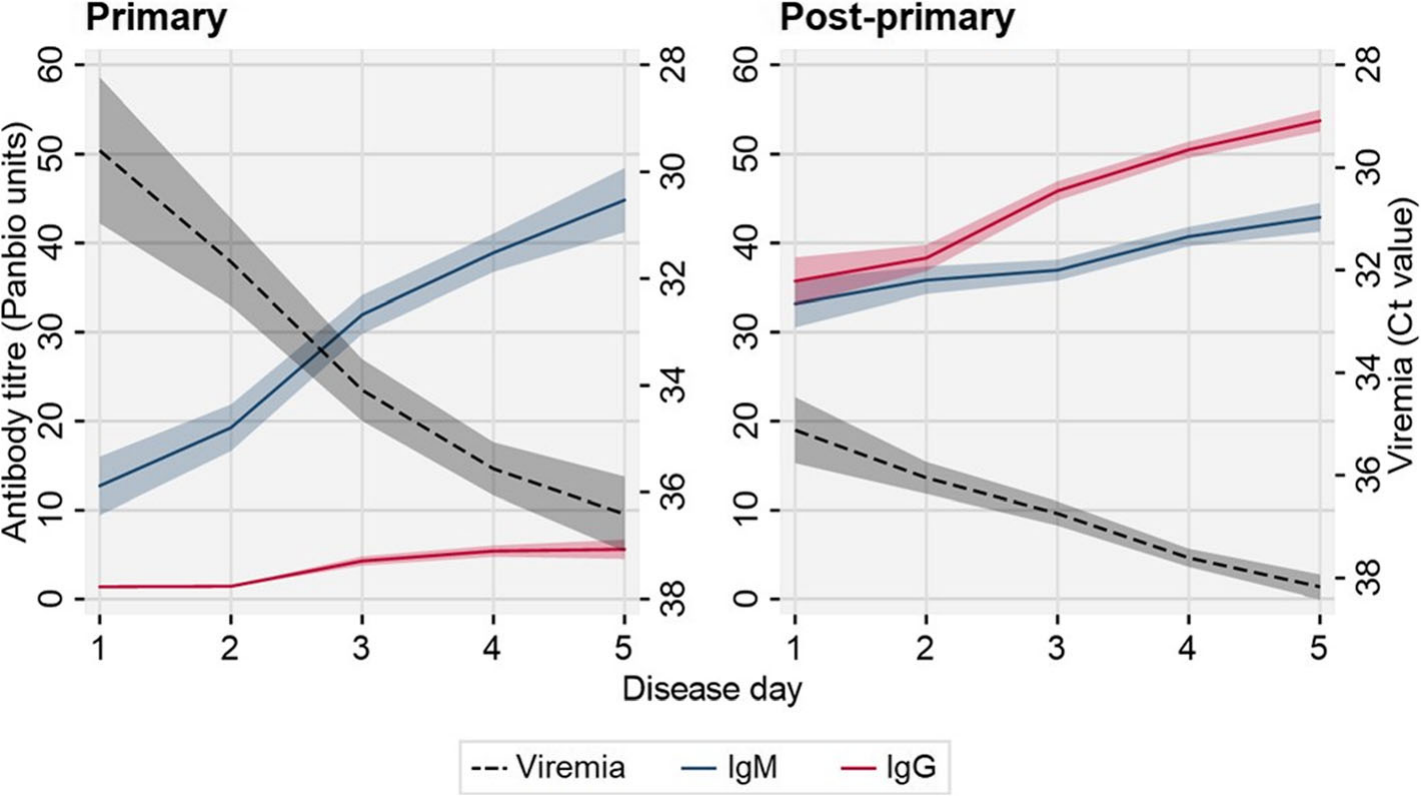
Primary and post-primary temporal infection kinetics. Disease day-specific, averaged, infection kinetics (Ct value, IgM and IgG panbio units) among acute active primary and post-primary dengue infections according to algorithm 2. Shading: 95% CI

To investigate the temporal kinetic infection patterns during acute primary and post-primary dengue infections, we calculated the mean anti-dengue viremia (Ct), IgM and IgG titres by disease day (Fig. 4). During the first 5 days of reported disease, mean IgM and IgG titres increased among both primary and post-primary infections, although IgG titres were very low among primary dengue infections. In contrast, mean dengue viremia decreased (increasing Ct) during the first 5 days of disease among primary and post-primary infections, although overall, this was significantly lower among post-primary infections.

**Fig. 5:**
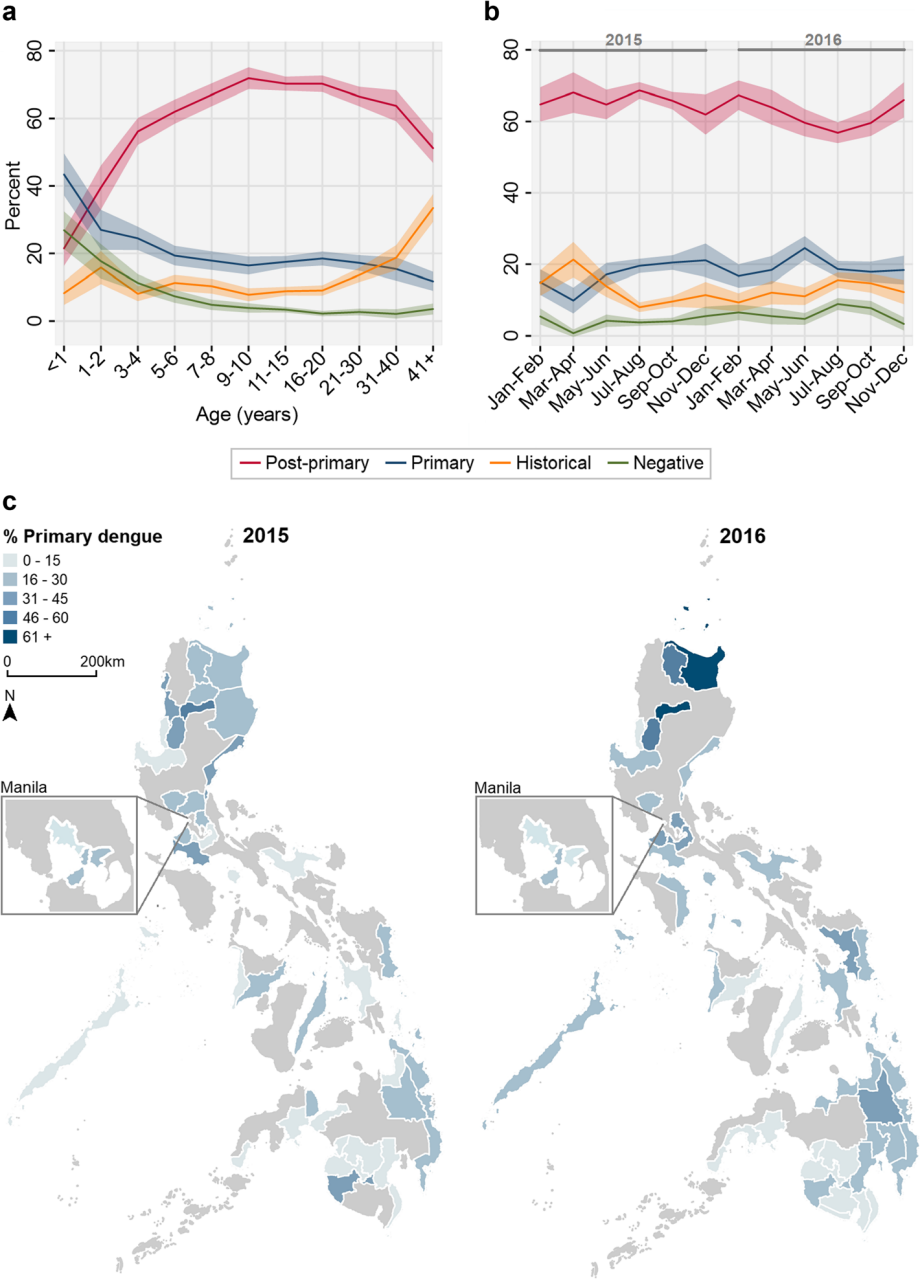
Immuno-epidemiological patterns associated with DENV immune status across the Philippines. **a** Percentage age trends in dengue immune status among the study population. Shading: 95% CIs. **b** Bi-monthly percentage trends in dengue immune status among the study population between 2015 and 2016. Shading: 95% CIs. **c** Provincial spatio-temporal percentage trends in primary dengue immune status among active DENV cases reporting across the Philippines between 2015 and 2016. Provinces with less than 10 samples collected excluded. Dengue immune status determined according to algorithm 2

Among active dengue cases, we investigated potential risk factors associated with post-primary compared to primary dengue status (Table 2). Individual risk factors included age (31+ compared to 0–5 years: OR 1.91 [1.54–2.38], *p* value < 0.001) and presenting with severe symptoms (severe compared to no warning signs: OR 1.66 [1.28–2.16], *p* value < 0.001) or warning signs (warning signs compared to no warning signs: OR 1.47 [1.28–1.70], *p* value < 0.001). DRU-level risk factors include decreasing ground elevation (150+ compared to 0–75 m: OR 0.61 [0.52–0.72], *p* value < 0.001) and increasing population density (200+ compared to 0–100 km^2^: OR 1.32 [1.14–1.53], *p* value < 0.001) consistent with the known epidemiology of dengue transmission. The strong univariate association between post-primary dengue and age prompted us to explore fine-scale age trends with dengue immune status. According to percentage trends, those aged between 0.5 and 1 year with active dengue infections were more likely to be primary, rather than post-primary dengue cases. After which, the percentage of those reporting with primary dengue decreased with age while post-primary dengue cases increased, plateaued then decreased with age. Among non-active dengue cases, the percentage reporting with negative and historical dengue were mainly younger and older, respectively (Fig. 5a).

**Table 2:** Risk factors associated with post-primary, opposed to primary, active dengue immune status

Risk factor	Post-primary		
	OR	95% CI	*p* value
**Age**			
< 5	1		
6–15	1.69	1.46–1.96	< 0.001
16–30	1.80	1.53–2.11	< 0.001
> 31	1.91	1.54–2.38	< 0.001
**Sex**			
Female	1		
Male	0.94	0.85–1.04	0.248
**Disease day**			
1–2	1		
3–4	1.06	0.94–1.19	0.377
5	1.00	0.84–1.19	0.973
**Clinical manifestation**			
No warning signs	1		
Warning signs	1.47	1.28–1.70	< 0.001
Severe	1.66	1.28–2.16	< 0.001
Non-disclosed	0.90	0.76–1.07	0.227
**DRU elevation (metres)**			
0–75	1		
75–150	0.81	0.67–0.97	0.023
150+	0.61	0.52–0.72	< 0.001
**DRU pop den (km**^**2**^**)**			
0–100	1		
100–200	0.87	0.77–0.97	0.017
200+	1.32	1.14–1.53	< 0.001

*OR* unadjusted odds ratio, *Pop den* population density, *DRU* disease-reporting unit

**Fig. 6:**
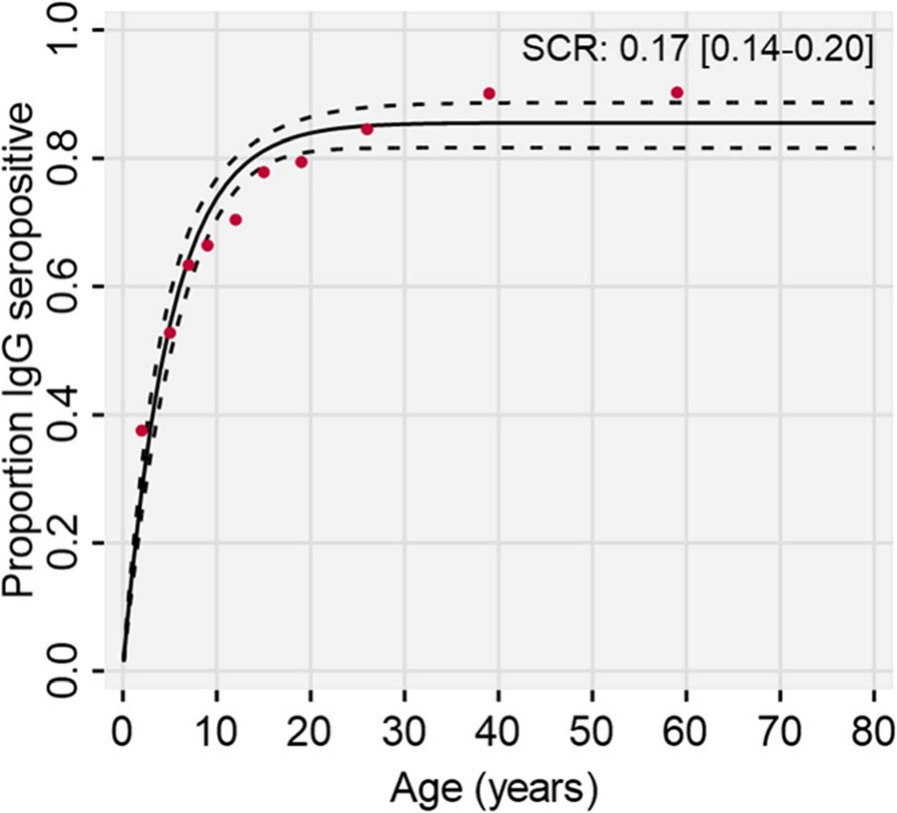
Dengue transmission intensity among non-active dengue cases across the Philippines between 2015 and 2016. Red dot: observed age-IgG seroprevalence. Black solid line: predicted age-IgG seroprevalence according to reverse catalytic model. Black dash line: predicted age-IgG seroprevalence 95% CI. SCR, seroconversion rate [95% CI]

Lastly, we explored spatio-temporal trends of dengue transmission dynamics across the Philippines during 2015 and 2016. Upon investigating dengue transmission intensity across the country, we revealed a reversible versus a simple, catalytic model best fits the age-seroprevalence data among reporting non-active dengue cases (Lrtest *p* value < 0.001). Using this statistically favoured model, we estimated a seroconversion rate of 0.17 [95% CI 0.14–0.20] among all non-active dengue reported cases (Fig. 6). Assuming individuals seeking care are representative of the general population, this suggests that 17% of the population were exposed to dengue annually. Additionally, bi-monthly percentage trends revealed temporal stability in the immune status of the reporting population across the Philippines between 2015 and 2016, with the majority reporting being post-primary cases (Fig. 5b). Despite this, we observed spatio-temporal heterogeneity in the immune status of the reporting population at lower administrative levels. In northern Luzon provinces during 2016, a higher percentage of primary cases reported compared to the rest of the Philippines (Fig. 5c).

## Discussion

In this study, we generated and validated a novel algorithm capable of distinguishing primary and post-primary immune status among reporting, suspected dengue cases during the first 5 days of fever using a single serum sample. By incorporating molecular and serological metrics, redefining dengue antibody exposure and using IgG:IgM ratios at appropriate stages of infection, we were able to propose a dengue immune status algorithm that was superior to existing practice. Subsequently, we demonstrated how the algorithm can be applied for dengue surveillance purposes across the Philippines. We revealed that post-primary dengue cases, who are at higher risk of progressing to severe outcomes, appear to be older than primary infections and were more likely to report to health facilities in low lying, urban areas. In addition, we showed primary dengue infections, who are at risk of subsequent post-primary infections in future years, spatially clustered around the northern regions of the Philippines in 2016.

According to a solely serological commercial immune status algorithm, a large percentage of the study population were unclassifiable. This shortcoming was overcome by incorporating individual molecular metrics into our novel algorithm, which captured early stage, non-immunogenic, primary dengue infections. Our algorithm also redefined seroprevalence to IgM and IgG using mixture modelling. We questioned whether febrile primary infections could exceed the standard IgG threshold, as previously demonstrated [16], and were concerned with determining the immune status of non-active dengue cases that may have elevated anti-DENV IgG following previous dengue exposure. Upon redefining serological exposure to anti-DENV IgM and IgG, we classified those with active dengue infections as being either IgM or PCR positive given both rise and fall respectively during an active dengue infection [4, 5]. As dengue IgM persists for months following infection [4, 29], it could be argued our algorithm categorises recent dengue infections as active infections. However, elicited IgM provides temporary immunity to other serotypes and the study population included those seeking healthcare, so we considered it unlikely for individuals to seek treatment with dengue-like symptoms for a past infection. In this study, antibody seroprevalence corresponded to the lowest panbio units with > 50% probability of being seropositive according to mixture models. As a result, some individuals with panbio units close to the generated thresholds may have been misclassified. However, given the two-tiered nature of our immune status algorithm, thresholds offer the most practical solution for categorising the study population.

We used disease day-specific IgG:IgM ratios to characterise primary and post-primary dengue status as day of fever is a common variable in dengue surveillance worldwide. As anti-DENV IgG is absent or very low during febrile primary infections and pre-circulates in post-primary infections due to previous dengue exposure [17, 29, 30], we concluded the observed lower and higher IgG:IgM ratio distributions represented primary and post-primary cases, respectively. Interestingly, early during the febrile infection period, there was no statistical justification for the existence of two ratio distribution peaks, so we refrained from using antibody ratios before disease day 3. This is consistent with previous findings, which state antibody ratios are poor determinants of immune status early during infection, likely due to low antibody responses [16]. Instead for very early stage dengue infections, we opted to use our newly generated IgG exposure threshold to assess primary and post-primary dengue given the delay in eliciting anti-DENV IgG during primary infections. Together, these findings suggest that the combination of IgG seroprevalence and IgG:IgM ratio thresholds, at appropriate stages of infection, is desirable for distinguishing dengue immune status among febrile reporting cases. In our study, we adhered to manufacturers’ specifications to ensure our algorithm is compatible for dengue surveillance operations elsewhere. However, improvements in assay performance, including antibody avidity estimates, may further enhance this immune status algorithm.

Compared to a commercial algorithm, our dengue immune status algorithm had a stronger serological agreement with the WHO gold standard method [22], which demonstrated its suitability for dengue surveillance and epidemiological analysis. It should be noted, however, that observed serological discordance between our novel algorithm (A2) and the WHO gold standard may be attributed to temporal changes in dengue infection status. Individuals categorised as negative or historical according to A2 could be infected with dengue between the paired sera interim and therefore be classified as primary or post-primary, respectively, based on WHO criteria. Overall, based on the short interval between acute and convalescent dengue sera collections and the suitability of A2, we found substantial agreement between A2 and the WHO gold standard method.

In our study, we found a significant proportion of post-primary dengue infections had serological evidence of historical, yet not recent, ZIKV exposure. This supports the hypothesis that other, structurally homologous flaviviruses, including ZIKV, elicit IgG responses that serologically prime individuals for subsequent post-primary, instead of primary, dengue infections. A finding previously reported [31, 32]. However, due to unknown specificities [23–25], we cannot exclude the possibility commercial ELISA kits are detecting antibodies elicited from more than one type of flavivirus. Therefore, we assumed that post-primary dengue infections may have been preceded by any flavivirus infection. Determining whether cross-reactive antibody responses are attributed to just one or both Zika and dengue infections remains an area of ongoing investigation.

Following the immune status classification of our study population, we reported contrasting disease day-averaged infection kinetics among primary and post-primary dengue cases consistent with previous studies [16, 29, 33]. The observed, lower viremia during the acute stage of post-primary, compared to primary, dengue infections has been previously reported [34, 35]. We also revealed the majority of the reporting population were post-primary dengue cases, as previously reported in the Philippines [36], and likely a consequence of the higher risk of more severe symptoms prompting more to seek healthcare. We found lower ground elevation and higher population density as risk factors for reporting with post-primary infections among active dengue cases. This is consistent with the rationale of favourable mosquito breeding conditions in lower (warmer) altitudes and areas of high human population density promote mosquito populations [37] and increase dengue transmission intensity. However, geographical imbalances in disease awareness and healthcare access, which we were unable to adjust for in this study, may also influence this association. Together with the serological validation, these immuno-epidemiological patterns provided further evidence our algorithm accurately characterised the immune status of the study population.

Between 2015 and 2016, we estimated that 17% of our study population became serologically exposed to dengue annually, which is consistent with the previous estimated force of infection between 11 and 22% generated in Cebu, central Philippines, in 2016 [36]. However, given these cases passively reported, it could be speculated those reporting with non-dengue fever were more likely to seek treatment if they had previous dengue infection(s) due to heightened symptom awareness. Therefore, our estimates are likely a slight overestimation of true dengue transmission intensity across the Philippines. Moreover, spatio-temporal heterogeneity in dengue transmission intensity [38–41] infers this national estimate is unlikely to be representative of lower administrative areas in the Philippines. Among those reporting with active dengue, dengue immune status remained temporally stable across the country yet spatially heterogeneous in northern Luzon. The northern cluster of increased primary dengue reporting was possibly attributed to recent dengue emergence, previously shown in Mexico [42], and/or above average healthcare access/disease awareness. Either way, these reflect populations at risk of developing post-primary infections following a novel serotype invasion. Such areas may also be worth targeting for control and/or enhanced disease surveillance.

## Conclusion

In this study, we constructed a framework to accurately categorise the dengue immune status of a large reporting population of suspected dengue cases across the Philippines using routinely collected surveillance metrics. Using our algorithm, we were able to investigate detailed dengue transmission dynamics over 2 years and revealed target populations at risk of developing severe disease. It is hoped that laboratory surveillance operations, in the Philippines and elsewhere, can apply our framework to monitor primary and post-primary infection epidemiology and inform targeted dengue control.

## Supplementary Information

**Additional file 1.** Stratification flow chart of surveillance data used in this study. Exclusion steps associated with the final dataset used in this study.

**Additional file 2.** Study population demographics. Demographic characteristics of study population with complete data (Final dataset), those missing serological /molecular data and those missing symptom data.

**Additional file 3.** Anti-DENV IgM mixture model component selection. Model fit comparison of a 3-component, compared to a 1-component, mixture model characterising the anti-DENV IgM titre distribution of the study population. AIC: Akaike information criterion.

**Additional file 4.** Age-stratified anti-DENV IgM and IgG panbio units. (**A**) Age-stratified anti-DENV IgM distributions of the study population fitted with 3-component mixture models. Black dash: Lowest IgM panbio unit with a classification probability of being seropositive>seronegative (0-5 years: 9.8, 6-15 years: 10.1, 16-30 years: 10.3, 31+ years: 9.7). (**B**) Age-stratified anti-DENV IgG distributions of non-active DENV cases fitted with 2-component mixture models. Black dash: Lowest IgG panbio unit with a classification probability of being seropositive>seronegative (0-5 years: 2.0, 6-15 years: 2.2, 16-30 years: 2.4, 31+ years: 2.3).

**Additional file 5.** Anti-DENV IgG mixture model component selection. Model fit comparison of a 2-component, compared to a 1-component, mixture model characterising the anti-DENV IgG titre distribution of non-active DENV cases. AIC: Akaike information criterion.

**Additional file 6.** Anti-DENV IgG:IgM mixture model component selection. Model fit comparison of 2-component, compared to 1-component, mixture models characterising disease day stratified IgG:IgM ratio distributions among active DENV cases. AIC: Akaike information criterion. Bold: statistically favoured model component.

**Additional file 7.** Validation of A2 compared to the WHO gold standard method of determining dengue immune status. WHO immune classification: dengue immune status according to WHO guidelines. Blue: serological agreement. Red: Serological disagreement.

**Additional file 8.** Validation of A1 compared to the WHO gold standard method of determining dengue immune status. WHO immune classification: dengue immune status according to WHO guidelines. Blue: serological agreement. Red: Serological disagreement.

**Additional file 9.** Scatter plots of anti-DENV and anti-ZIKV IgM (blue) and IgG (red) among those categorised as primary and post-primary dengue according to A2. Horizontal dash: seroprevalence thresholds according to Euroimmune™ specifications (1.1 antibody ratios).

## Data Availability

The datasets used in this study are available from the corresponding author, on reasonable request, following approval from appropriate institutional committees.

## Abbreviations

ADE: Antibody-dependent enhancement
AIC: Akaike information criterion
CI: Confidence interval
Ct: Cycle threshold
DENV: Dengue virus
DOH: Department of Health
DRU: Disease-reporting unit
ELISA: Enzyme-linked immunosorbent assay
GPS: Global positioning system
HIA: Haemagglutination inhibition assay
IgG: Immunoglobulin G
IgM: Immunoglobulin M
OR: Odds ratio
PCR: Polymerase chain reaction
PIDSR: Philippine Integrated Disease Surveillance and Response
RDT: Rapid diagnostic test
RNA: Ribonucleic acid
SCR: Seroconversion rate
WHO: World Health Organization
ZIKV: Zika virus
