# Supplementary figures and images for: A serological framework to investigate acute primary and post-primary dengue cases reporting across the Philippines
Source: BMC Med. 2020 Nov 27;18:364. doi: 10.1186/s12916-020-01833-1 (PMC7694902; doi:10.1186/s12916-020-01833-1)

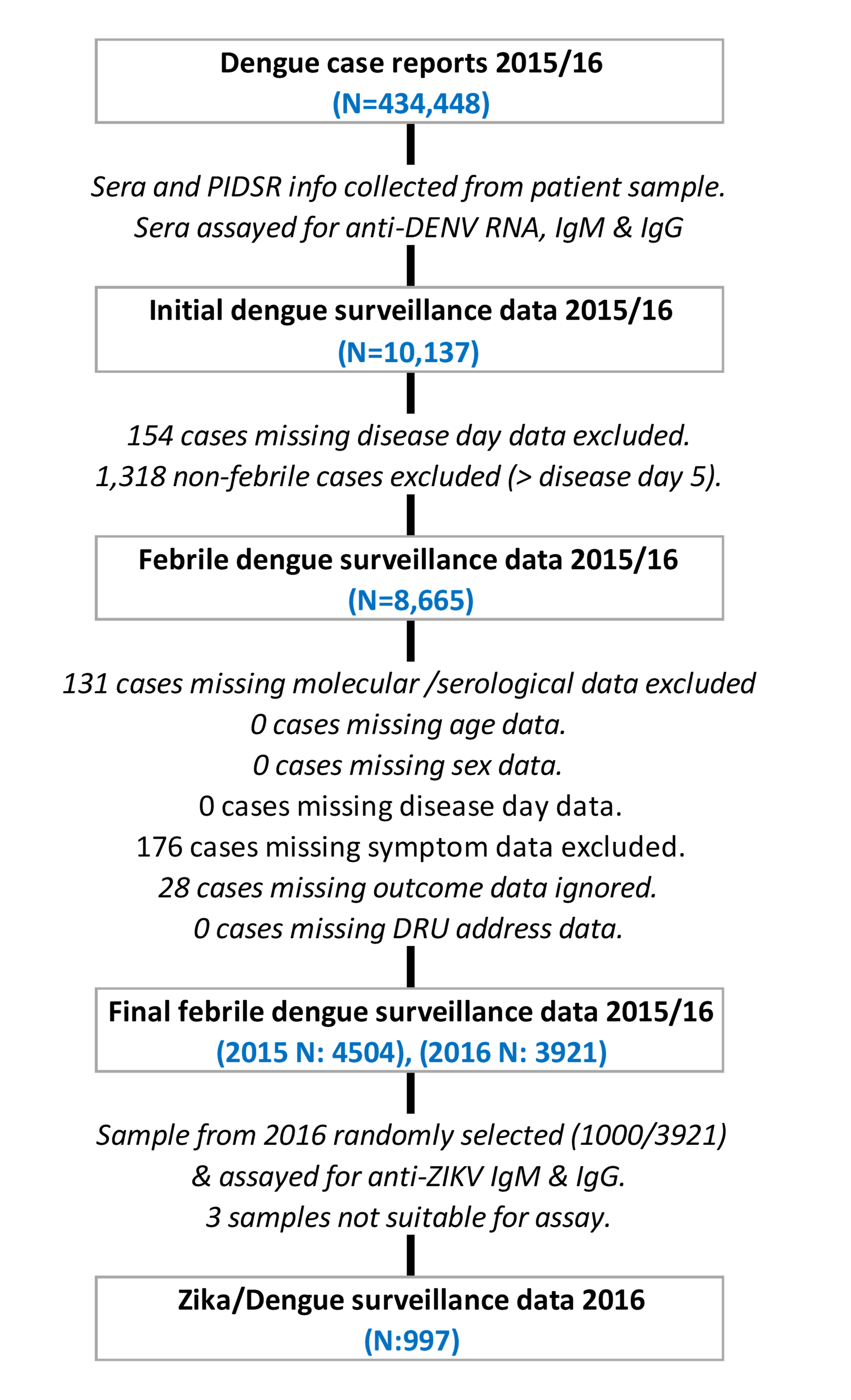

Supplement: Supplementary file 1 — Additional file 1. Stratification flow chart of surveillance data used in this study. Exclusion steps associated with the final dataset used in this study. [file 12916_2020_1833_MOESM1_ESM.tif]

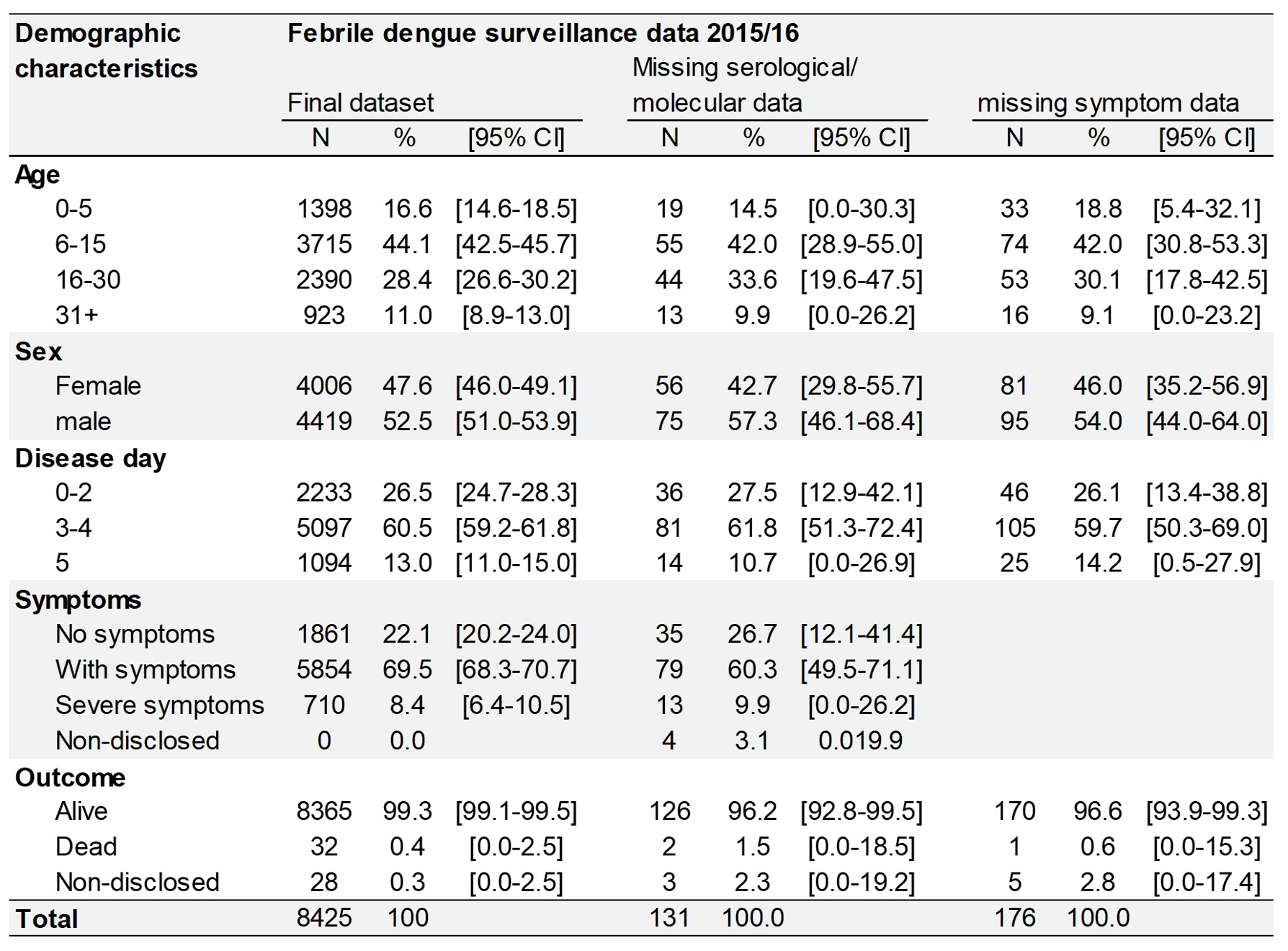

Supplement: Supplementary file 2 — Additional file 2. Study population demographics. Demographic characteristics of study population with complete data (Final dataset), those missing serological /molecular data and those missing symptom data. [file 12916_2020_1833_MOESM2_ESM.tif]

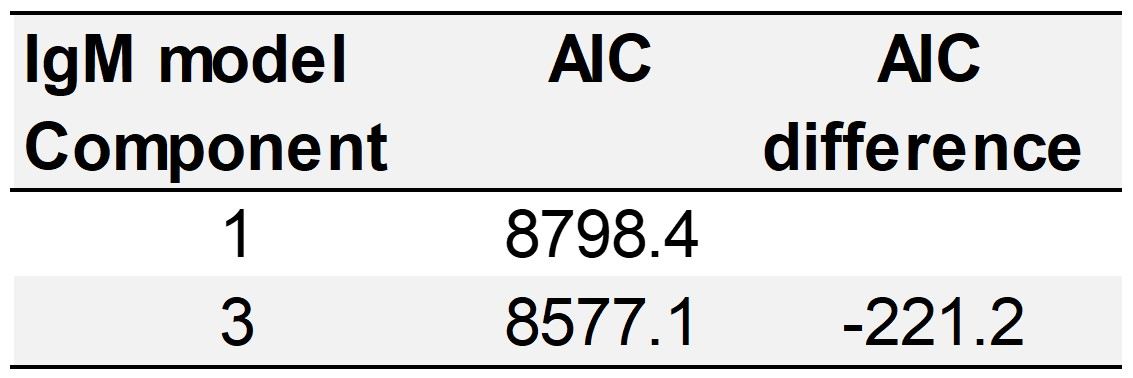

Supplement: Supplementary file 3 — Additional file 3. Anti-DENV IgM mixture model component selection. Model fit comparison of a 3-component, compared to a 1-component, mixture model characterising the anti-DENV IgM titre distribution of the study population. AIC: Akaike information criterion. [file 12916_2020_1833_MOESM3_ESM.tif]

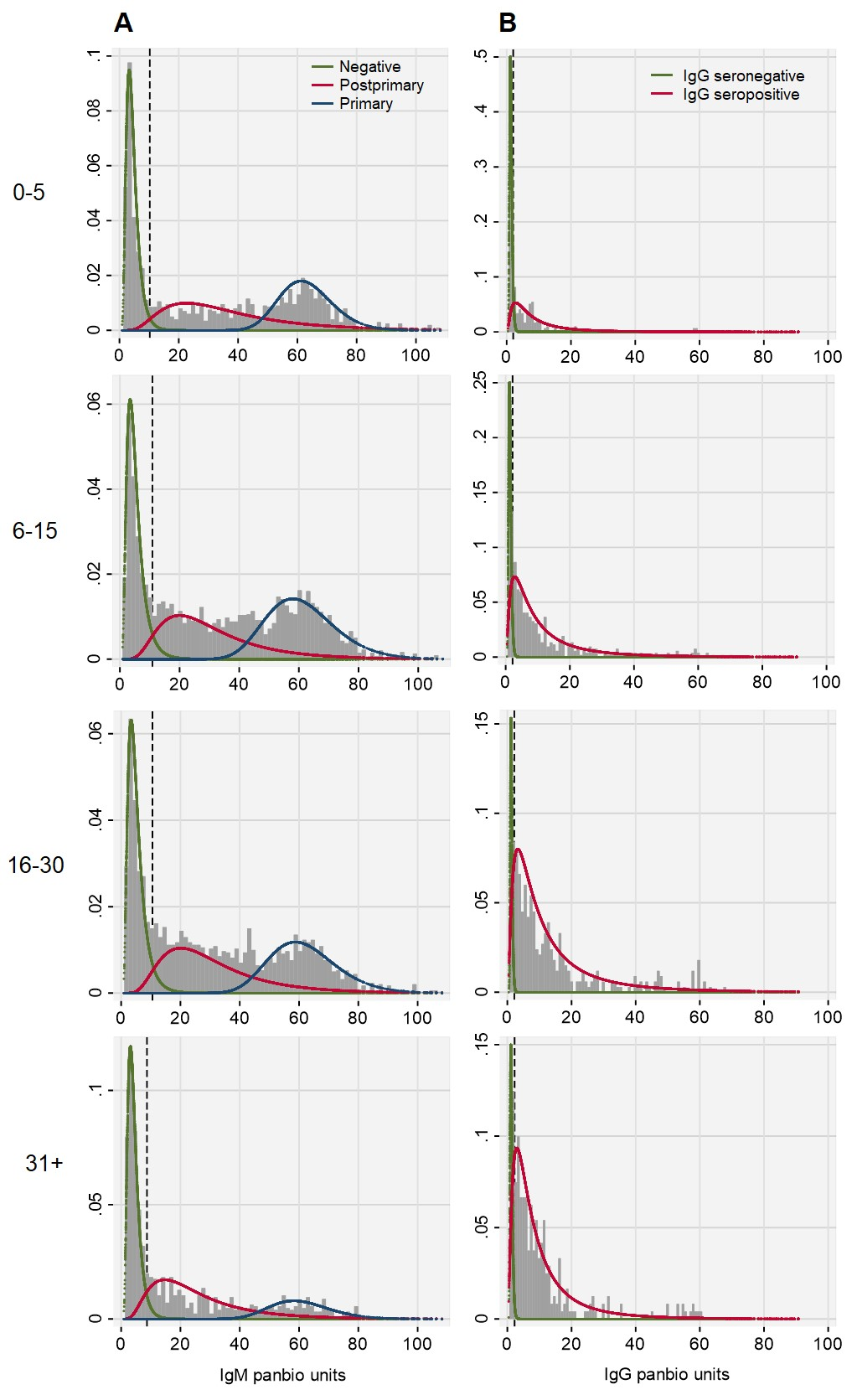

Supplement: Supplementary file 4 — Additional file 4. Age-stratified anti-DENV IgM and IgG panbio units. (A) Age-stratified anti-DENV IgM distributions of the study population fitted with 3-component mixture models. Black dash: Lowest IgM panbio unit with a classification probability of being seropositive>seronegative (0-5 years: 9.8, 6-15 years: 10.1, 16-30 years: 10.3, 31+ years: 9.7). (B) Age-stratified anti-DENV IgG distributions of non-active DENV cases fitted with 2-component mixture models. Black dash: Lowest IgG panbio unit with a classification probability of being seropositive>seronegative (0-5 years: 2.0, 6-15 years: 2.2, 16-30 years: 2.4, 31+ years: 2.3). [file 12916_2020_1833_MOESM4_ESM.tif]

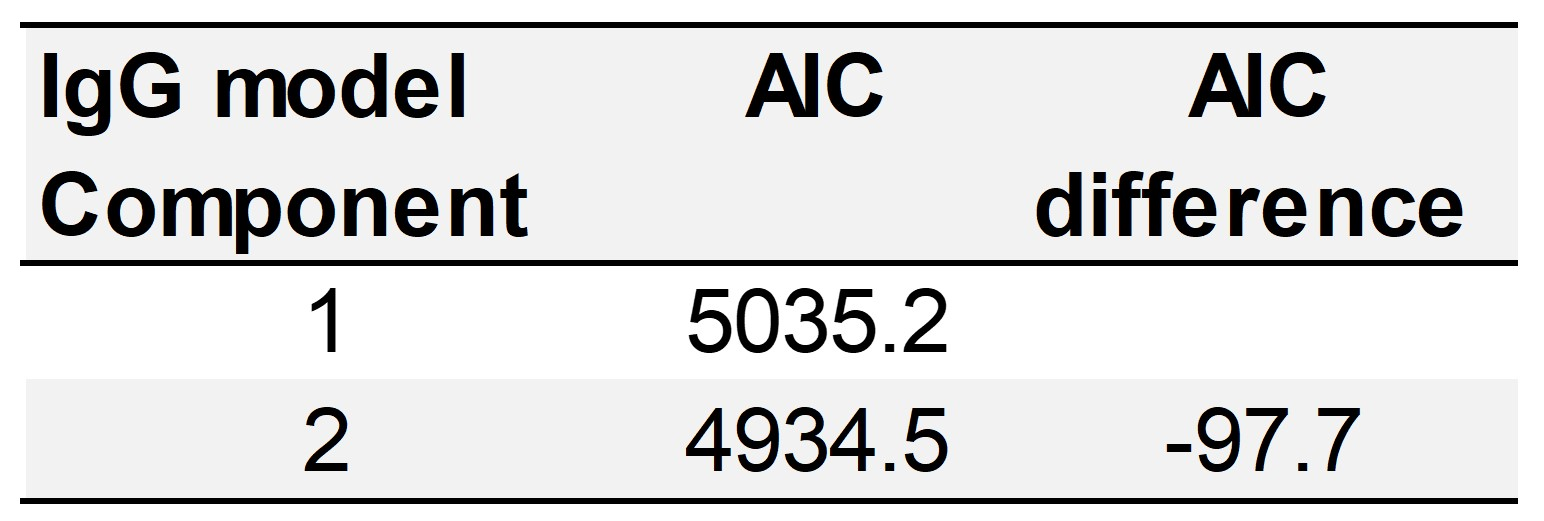

Supplement: Supplementary file 5 — Additional file 5. Anti-DENV IgG mixture model component selection. Model fit comparison of a 2-component, compared to a 1-component, mixture model characterising the anti-DENV IgG titre distribution of non-active DENV cases. AIC: Akaike information criterion. [file 12916_2020_1833_MOESM5_ESM.tif]

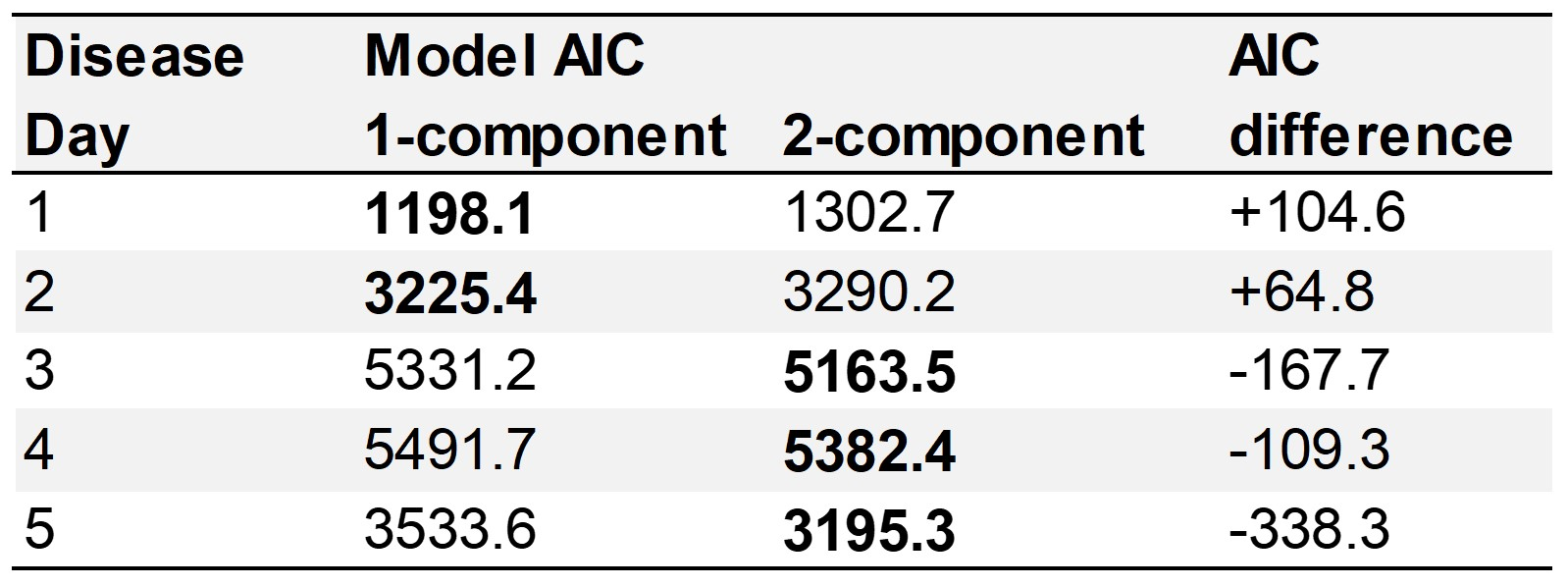

Supplement: Supplementary file 6 — Additional file 6. Anti-DENV IgG:IgM mixture model component selection. Model fit comparison of 2-component, compared to 1-component, mixture models characterising disease day stratified IgG:IgM ratio distributions among active DENV cases. AIC: Akaike information criterion. Bold: statistically favoured model component. [file 12916_2020_1833_MOESM6_ESM.tif]

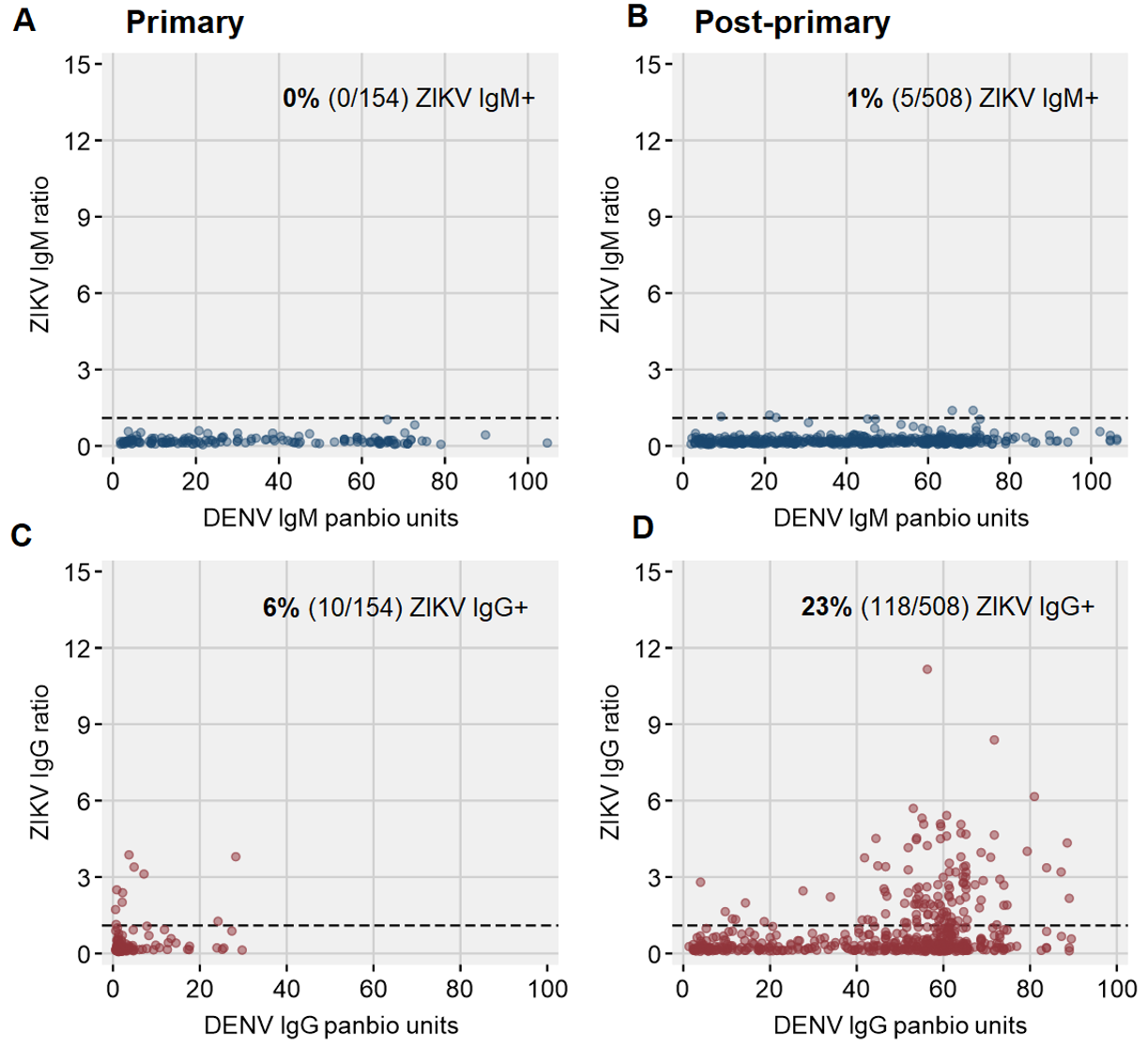

Supplement: Supplementary file 9 — Additional file 9. Scatter plots of anti-DENV and anti-ZIKV IgM (blue) and IgG (red) among those categorised as primary and post-primary dengue according to A2. Horizontal dash: seroprevalence thresholds according to Euroimmune™ specifications (1.1 antibody ratios). [file 12916_2020_1833_MOESM9_ESM.tif]
